# Meningitis due to *Haemophilus influenzae* type f

**DOI:** 10.1590/S1679-45082013000400020

**Published:** 2013

**Authors:** Marta Pessoa Cardoso, Jacyr Pasternak, Alfredo Elias Giglio, Rejane Rimazza Dalberto Casagrande, Eduardo Juan Troster

**Affiliations:** 1 Hospital Israelita Albert Einstein, São Paulo, SP, Brazil; 2 Instituto da Criança, Hospital das Clínicas, Faculdade de Medicina, Universidade de São Paulo, São Paulo, SP, Brazil

**Keywords:** Haemophilus influenzae, Meningitis/diagnosis, Brazil, Case reports

## Abstract

With the decline in the rate of infections caused by *Haemophilus influenzae* serotype b since the widespread vaccination, non-b serotypes should be considered as potential pathogenic agents in children with invasive disease younger than 5 years old. We report the case of an immunocompetent 1-year-old boy with *Haemophilus influenzae* type f meningitis. The agent was identified in cerebrospinal fluid and blood cultures. Serotyping was performed by tests using polyclonal sera and confirmed by polymerase chain reaction. All *Haemophilus influenzae* isolates associated with invasive disease should be serotyped and notified as a way to evaluate the changes and trends in serotype distribution of this disease.

## RESUMO

Com o declínio da taxa de infecções causadas pelo *Haemophilus influenzae* tipo b após a ampla introdução da vacina, sorotipos não-b devem ser considerados agentes patogênicos potenciais em crianças menores de 5 anos com doença invasiva. Relatamos um caso de meningite por *Haemophilus influenzae* tipo f em um lactente imunocompetente de 1 ano. O agente foi identificado em líquido cefalorraquidiano e hemocultura. A sorotipagem foi realizada por testes com soros policlonais e confirmada por reação em cadeia de polimerase. Todas as cepas de *Haemophilus influenzae* associadas à doença invasiva deveriam ser sorotipadas e notificadas, a fim de possibilitar uma análise adequada das mudanças e tendências na distribuição de sorotipos desta doença.

## INTRODUCTION

*Haemophilus influenzae* (Hi) is a significant human pathogen whose encapsulated strains have been classified in serotypes a to f and nontypeable (NTHi) on the basis of the capsular polysaccharide. All these antigenic serotypes can cause invasive disease, which are most often reported in pediatric patients. Nonencapsulated strains are less virulent and rarely cause serious infection in children^(1)^.

Prior to the introduction of Hib conjugate vaccines in 1988, serotype b was responsible for more than 95% of all invasive Hi diseases worldwide. In the United States roughly 20,000 cases were diagnosed annually among children younger than 5 years old, presenting an incidence of about 100/100,000 people, which declined to 0.11 in 2010^(2)^. However, Hib remains a leading cause of meningitis among unvaccinated children especially in developing countries^(1)^.

In Brazil, Hib was also predominant during the pre-vaccine era (before 1999), being reported few cases on types other than b^(3–5)^. Data from a regional study of Hib meningitis showed an incidence of 25.4 per 100,000 of children population under 5 years of age, declining to 0.6 after 5 years of immunization^(3)^. Unfortunately, there are no official national data on the incidence of Hib and non-b invasive disease either before or after vaccine introduction in the country.

Widespread vaccination has raised the concern of serotype replacements, as other serotypes may fill the ecological niche that has been left open by the b type^(1,3)^. If this hypothesis is true, we should detect non-type b strains more often in the post-vaccine era.

As far as we know, three cases of *Haemophilus influenzae* type f (Hif) were reported in Brazil before 1999^(5)^. Since that year, 22 Hif strains were identified from patients with meningitis according to national reference laboratories^(4,5)^. We report here a case of an immunocompetent child diagnosed with Hif meningitis, and the reviewing of recent epidemiologic data from Brazil and other countries.

## CASE REPORT

A 1-year-old boy was admitted to the Pediatric Intensive Care Unit (PICU) at a private tertiary hospital in São Paulo, Brazil, with a history of fever in the last 3 days, vomiting in the last day before admission, and sleepiness in the last 12 hours. The patient had pneumonia 3 months before but nothing remarkable otherwise. His immunization schedule was complete. On admission, child temperature was 38.2°C/100.8°F, he was slightly pale and with prolonged capillary refill; he was irritable, alternating with lethargy, and presented signs of meningeal irritation. The child heart rate was 145bpm, and his blood pressure and oxygen saturation were normal. The cerebrospinal fluid (CSF) revealed 4,110 leukocytes/mm^3^ (83% neutrophils), protein of 440mg/dL, glucose under 20mg/dL and lactate of 107mg/dL. Latex agglutination test for *Haemophilus* spp. was inconclusive. The result of the computed tomography of the head was normal.

Ceftriaxone was given 30 minutes after admission. Peripheral perfusion improved after receiving 80mL/kg of normal saline. Cultures of the CSF and blood grew Hi identified as type f by the slide agglutination method with polyclonal sera. Serotype was confirmed by polymerase chain reaction (PCR)- based capsular genotyping using the Microseq system. Immunologic evaluation that included serum immunoglobulins and specific antibodies tests was normal.

The length of stay at PICU was 2 days and the child was discharged 12 days after admission. No hearing loss or neurodevelopmental disabilities were detected after the 5 years of follow-up.

## DISCUSSION

Systemic disease attributable to the serotype f is fatal in roughly 20% in children and 30% in adults. While most adult patients infected by these strains have significant underlying diseases, in children this condition is less common (26% of the cases). Pneumonia is the predominant clinical syndrome in adult patients with Hif invasive disease^(6)^. In children younger than 5 years old, pneumonia and meningitis are equally represented, but there are reports of unusual sites like endocarditis and septic arthritis in healthy children^(1,6)^. Recent history of upper respiratory tract infection preceding Hif infection is more common in children than in adults^(6)^.

In recent years, invasive disease caused by non-type b strains has been reported more frequently both in adults and children, but it is still unclear whether the absolute incidence of this disease has increased. Literature on the emergence of non-type b diseases is relatively scarce and shows heterogeneous results. Interestingly, there are geographic variations in the distribution of non-b invasive diseases, being serotypes a, e, f or NTHi more predominant in different regions^(1,5–9)^.

A multicenter study conducted in Canada during post-vaccine years showed that two-third of Hi invasive diseases were caused by non-b serotypes (mainly NTHi and Hia), with an average of 14.8 cases per year and that annual rate is increasing over the years. There is no data on the pre-vaccine incidence, as serotyping was not available at that time^(7)^. An active surveillance program in the United States reported an increase in proportion of invasive Hi disease caused by NTHi from 15% to 45% in 6 years (1989 to 1994), and by Hif from 1 to 17%, becoming close to that caused by Hib (18%). In this period, the absolute incidence of Hif cases rose from 0.5 to 1.9/1,000.000 people, whereas incidence of NTHi remained in 5.0/1,000.000 people^(6)^. However, more recently, data from Centers for Disease Control and Prevention stated that incidence of non-type b invasive infection as a whole remains stable in 0.8/100,000 children younger than 5 years in the United States^(2)^. In Europe, there was a small, but statistically significant, increase in invasive non-type b Hi disease, from 0.22 to 0.35/100,000 people in 10 years (1996 to 2006), according to an international surveillance project. In the under-5 age group, 75% of non-type b infections were caused by Hif, with an incidence of 0.78 cases per 1,000.000, followed by Hia and Hie infections^(8)^.

An investigation conducted in Latin America from 2000 to 2005 reported that Hib continues to be the most prevalent serotype (65%) in all group of ages under 14, followed by NTHi (24,7%), Hia (6,1%) and Hif (1,6%). However, the relative frequency of Hib is decreasing since 2000 and this decline could have been greater if the vaccine had been introduced simultaneously in all countries studied (it ranged from 1994 in Uruguay to 2006 in Guatemala). During the same period, the proportion of NTHi and Hia increased in children under 2, while Hif remained stable in all groups of age^(9)^.

In Brazil, a passive laboratory-based surveillance for Hi meningitis conducted from 1990 to 2008 showed decline in Hib isolates among children under 4, while identification of Hia and NTHi meningitis increased significantly among infants and children aged 1 to 4 years, respectively. In the post-vaccine period, Hib accounted for 59% of Hi meningitis isolates (blood or CSF); NTHi increased from 2 to 22% compared to the previous period, and non- b serotypes from 1 to 19%, of which 14% were Hia and 2,9% are Hif (21 cases)^(5)^. Despite the trend observed, the data did not suggest an absolute increase in non-b or NTHi invasive disease after Hib vaccination in Brazil or Latin America^(5,9)^.

Slide agglutination with polyclonal sera is the standard method for serotyping Hi. However, when compared to capsule typing by PCR or to monoclonal antibodies, that method with polyclonal sera has been shown to be the least reliable, with the concordance ratio between the standard method and the other two being only 76%^(10)^. In Brazil, tests with polyclonal sera still prevail. Therefore, this may impact the epidemiology of the different types in the country. Probably the occurrence of non-type b is even higher than presented here. Besides that, although Hi meningitis is subject to mandatory notification since 1977, it was not until 2000 that serotyping of all Hi isolates was recommended by the national health politics. This fact may impact the low incidence of non-type b Hi infections reported in the pre-vaccine era.

Changes over time and through different regions must be carefully interpreted, as they may reflect differences in surveillance, culturing practices, rate and method of serotyping, and underlying conditions of infected patients.

Increase of non-type b isolates or even a change in serotype distribution is a concern for public health surveillance, since no vaccines against these serotypes exist. Authors from mentioned studies suggest serotyping of all Hi isolates associated with invasive disease as a way to evaluate the vaccination program against type b, inform on the sensitivity of the surveillance system and identify invasive disease from types other than b^(2,5)^.

Serotype replacement has not been highly considered for Hib conjugate vaccines, probably because non-type b strains rarely used to cause invasive diseases. However, as presented here and by other studies, non-type b invasive disease has been observed more frequently in regions where the Hib conjugate vaccine has been used. Our report of Hif meningitis combined with the fact that there are few reports of its occurrence before 1999 in Brazil may contribute to the discussion of serotype replacement.

## CONCLUSION

All Hi isolates associated with invasive disease should be serotyped and notified as a way to evaluate the Hib vaccination program, inform on the sensitivity of the surveillance system and identify invasive disease from types other than b. Increase of non-type b isolates is a concern for public health surveillance, as no vaccines against these serotypes exist. Continued surveillance is warranted to evaluate the trend toward the increasing incidence of Hif and other non-type b disease, as has been discussed here and in other studies.
